# Perioperative Allogenenic Blood Transfusion is Associated With Worse Clinical Outcome for Patients Undergoing Gastric Carcinoma Surgery: A Meta-Analysis

**DOI:** 10.1097/MD.0000000000001574

**Published:** 2015-10-02

**Authors:** Lihong Li, Dajian Zhu, Xiaowu Chen, Yanfeng Huang, Manzhao Ouyang, Weijie Zhang

**Affiliations:** From the Department of General Surgery I, First People's Hospital of Shunde, Foshan City, Guangdong Province, China.

## Abstract

Whether perioperative allogenic blood transfusion (ABT) has adverse effect on patients with gastric carcinoma (GC) surgery or not, that is controversial. Our study evaluated the association between ABT and some clinical outcomes of GC surgery patients.

Data of relevant studies were based on PubMed, EMBASE, and the Cochrane Library search. The relative risk (RR) of 5-year survival rates, tumor recurrence, and postoperative complications were performed; subgroup analyses included district, transfusion rates, age, participants, sex, and tumor stage.

The study was approved by the ethics committee of the First People's Hospital of Shunde.

In total, 9189 participants from 16 studies were included in the meta-analysis. The 5-year survival rate was decreased for the GC patients with ABT (RR = 0.74, 95% confidence interval [CI] = 0.69–0.79), the risk of tumor recurrence was significantly higher for ABT patients (RR = 1.82, 95% CI = 1.32–2.51), and postoperative complications increased in ABT patients (RR = 1.36, 95% CI = 1.02–1.81), respectively; in subgroup analyses, 5-year survival rates were not associated with the transfusion rates (χ^2^ = 0.37, *P* = 0.54).

Transfusion for patients undergoing GC surgery, even low transfusion rates, would reduce the 5-year survival rates, and elevated the risk of tumor recurrence and postoperative complication.

## INTRODUCTION

Anemia is most common in gastric carcinoma (GC) patients, which may be caused by bleeding due to the necrosis of cancer, no appetite for food, lack of nutrition, and so on. Furthermore, after surgery for these patients, the iron absorption will become a problem because of short of intrinsic factor.

Transfusion is, however, a useful method to remedy anemia in patients with GC before and after surgery, which could cause lots of side effects, such as infectious disease including hepatitis B virus (HBV), human immunodeficiency virus (HIV), and so on; besides these virus disease, there are other complications, for example, iron overload, transfusion-related acute lung injury, hemolytic transfusion reaction, and transfusion-associated circulatory overload. Other studies have reported that transfusion not only caused such problems, but it could affect the GC surgery patients’ prognosis, such as elevating the recurrence rates of cancer, the rates of pertinent complications, and the mortality rates.^1^ Some reported that transfusion did not influence the long-term survival of patients with resected GC.^2,3^

So in order to clarify the inconsistent issue, a meta-analysis is necessary to be performed; the aim of our work was to inspect the association between transfusion and the 5-year survival rate, cancer recurrence, and complications in patients with GC undergoing surgery.

## SEARCH METHODS FOR IDENTIFICATION OF STUDIES

Electronic databases (PubMed, EMBASE, and the Cochrane Library) were searched to the October 31 in 2014 using the following search strings: “gastric cancer,” “stomach cancer,” “gastric carcinoma,” “stomach carcinoma,” “gastric neoplasm,” “stomach neoplasm,” and “transfusion.” The citations to be searched were restricted to human studies and published in English. Duplicates and out of scope publications were excluded. Some relevant lists were searched manually for additional studies missed by the search strategy. The full text of the remaining publications was screened for eligibility for data extraction and meta-analysis.

### Inclusion Criteria

In this meta-analysis, the citations should meet the following criteria: evaluation of the association between allogenic blood transfusion (ABT) and clinical prognosis (postoperative complications, recurrence, or 5-year survival rate) for patients with GC surgery; studies were excluded if the report focus on autogenic blood transfusion or benign tumors or no surgery; it must be clinical studies, not experimental studies on animals; and the specific data could be obtained.

If duplicate studies were obtained, we chose the latest one in this meta-analysis. However, if studies could provide additional information, that would be included in the subgroup analyses of this article.

### Data Extraction

Potentially relevant articles were identified by 2 reviewers (LL and DZ) with the predefined search methods and included in this meta-analysis according to the criteria above. Participants’ information was abstracted and listed in Table 1 by 2 authors (YH and WZ). If discrepancies were emerged, that would be resolved through discussion in all of the authors till consensus was achieved.

**TABLE 1:**
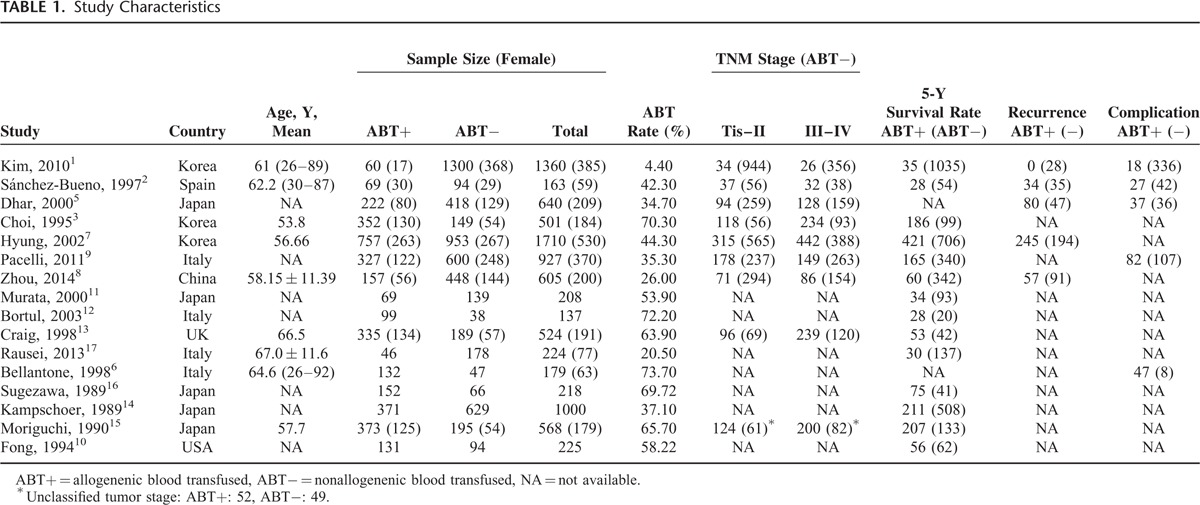
Study Characteristics

### Data Synthesis and Statistical Analysis

In this meta-analysis, all participants were divided into 2 groups (ABT group and non-ABT group). If patients were received any kind or amount of blood transfusion, that were included in the ABT group, and if not received any blood products, that were classified into non-ABT group. Participants’ information contained first author's name, publication year, patients’ age and sex, number of patients included in the ABT and non-ABT group, transfusion rate, tumor stage, survival data, tumor recurrence, and complication for patients with and without ABT, respectively.

The relative risk (RR) of 5-year survival rate, cancer recurrence rate after surgery, and postoperative complication associated with ABT were considered 3 main outcomes in this article, and subgroup analyses were district (Asian vs non-Asian), transfusion rates (<50% vs ≥50%), age (<60 years v. ≥60 years), participants (<600 vs ≥600), sex (male vs female), and tumor stage (Tis–II vs TNM III–IV).

χ^2^ and I^2^ statistics were used to test heterogeneity (values of 25%, 50%, and 75% were considered to represent low, medium, and high heterogeneity, respectively).^4^ If the heterogeneity of any outcome was >75%, then the subgroup was conducted to evaluate the potential factor that may lead to the heterogeneity. For each outcome, RR and its 95% confidence intervals (CIs) were used to measure the association for each study. Multivariate regression model was used to analyze the confounding factor, to determine whether transfusion was an independent risk factor on prognosis of GC patients after surgery. All data analyses were conducted by RevMan software version 5.1 (The Nordic Cochrane Centre, Rigshospitalet, Copenhagen, Denmark) and Stata SE version 12.0 software (Stata Corp. LP, College Station, TX).

In addition, sensitivity analyses were carried out by excluding a highest or lowest RR value to assess the stability of the association between ABT and cancer clinical outcomes. *P* values were 2-tailed and the statistical significance was set at 0.05.

## RESULTS

### Selected Studies and Characteristics

The selection of studies for inclusion in the meta-analysis was shown in Flow diagram. Of the initial 502 citations retrieved, our final primary analysis included 16 articles with a total of 9189 participants (2 articles^5,6^ [the participants were 640 and 179, respectively] did not include the concrete data of 5-year survival rate but tumor recurrence and complications, so we incorporated them into the meta-analysis). Five articles contained tumor recurrence data^1,2,5,7,8^ and complications,^1,2,5,6,9^ so the outcomes were performed by the correlated data.

Detailed study characteristics were shown in Table 1.^1–3,5–17^ Of the 16 studies, all of which were prospective cohort studies, 9 were from Asia (1 from China, 5 from Japan, and 3 from Korea), 4 from Italy, and 1 each from the United States, the United Kingdom, and Spain. The proportion of Asians was 57.3% (n = 5263). In total, 9189 GC patients undergoing surgery were included, of whom 4117 (44.8%) received an ABT, and the sample size ranged from 137 to 1710 (46–757 in ABT group and from 38 to 1300 in non-ABT group). The follow-up duration ranged from 1.5 to 22 years. Outcomes reported in each study included 5-year survival rate (n = 14), tumor recurrence (n = 5), and postoperative complications (n = 5). The results of subgroup analysis are shown in Table 2 and Table 3, and the results of survival rate after adjustment of covariates in this meta-analysis are shown in Table 4.

**TABLE 2:**
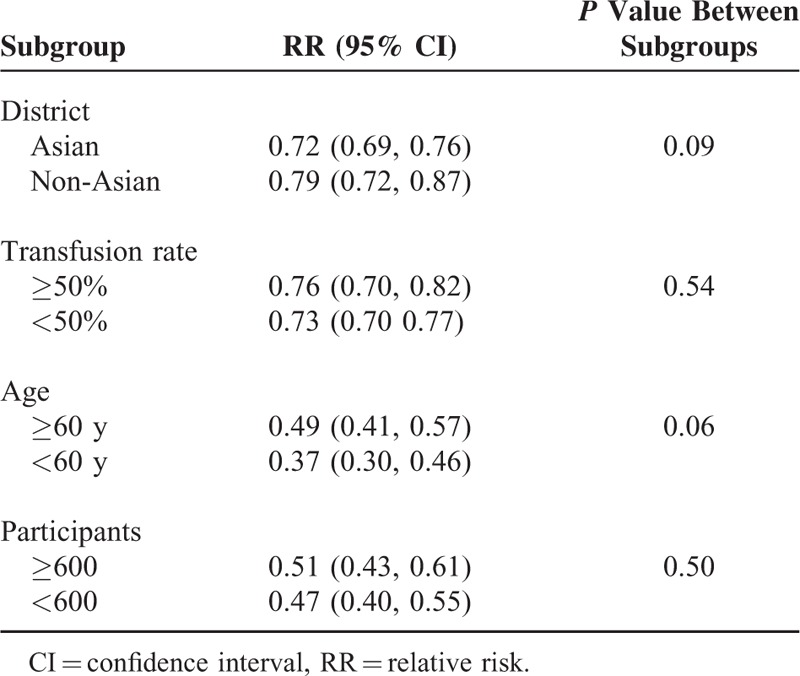
Subgroup Analysis of the Association Between Transfusion and 5-Year Survival Rate

**TABLE 3:**
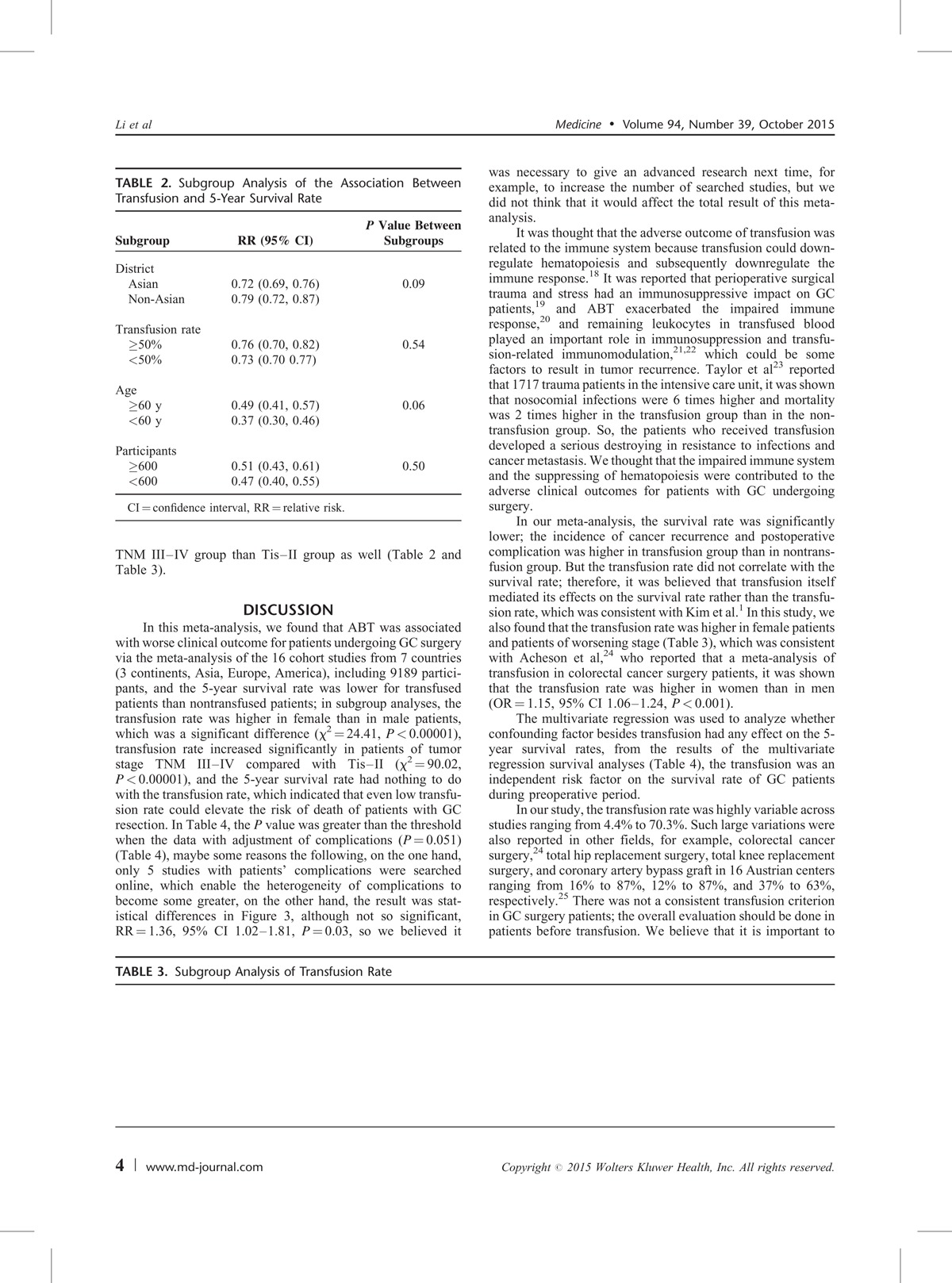
Subgroup Analysis of Transfusion Rate

**TABLE 4:**
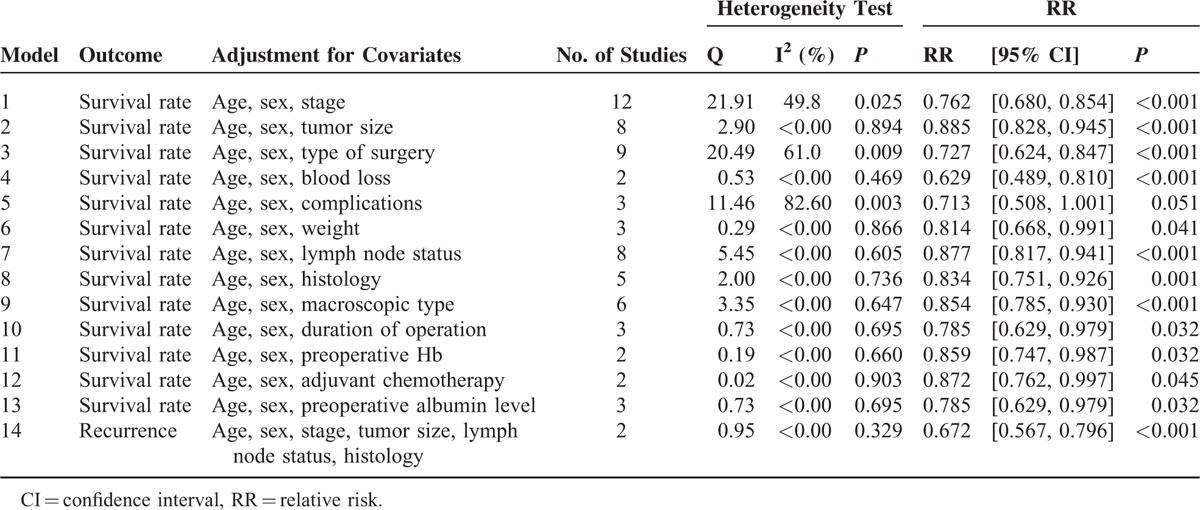
The Meta-Analysis Results of Survival Rate After Adjustment of Covariate

## OUTCOMES

### Survival Rate

**FIGURE 1:**
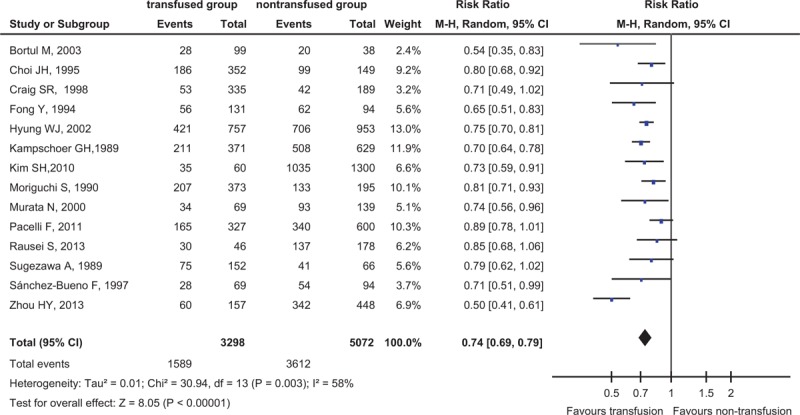
Forest plot of comparison: transfusion vs nontransfusion: outcome: 5-year survival rate. Answer: The concrete legends were shown in last paragraph of page 6.

The data were heterogeneous (I^2^ = 58%), so we used the random-effects model to combine results from all studies. Fourteen studies reported the 5-year survival rate (giving a total sample size of 8370 participants for evaluation), which was lower in ABT group (48.2%, 1589 cases in total of 3298 patients) than in nontransfused group (71.2%, 3612 in total of 5072 patients) (RR = 0.74, 95% CI 0.69–0.79, *P* < 0.00001) (Fig. 1).

To avoid the possibility of collinearity between ABT and other risk factors, we in turn pooled the data after adjusting series of covariates. As shown in our results (Table 4), ABT is a risk factor for survival rate independent from age, sex, stage, tumor size, type of surgery, blood loss, weight, lymph node status, histology, macroscopic type, duration of operation, preoperative Hb, adjuvant chemotherapy, and preoperative albumin level (RR < 1, *P* < 0.05).

### Cancer Recurrence Rate

**FIGURE 2:**
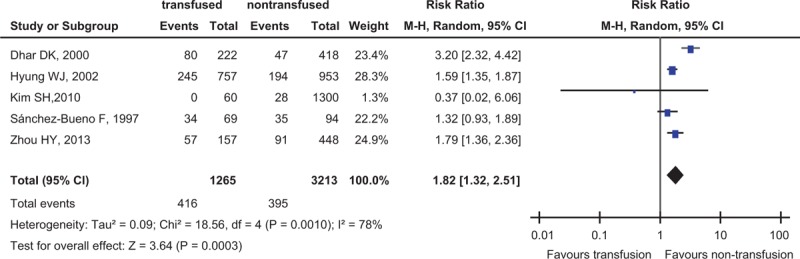
Forest plot of comparison: transfusion vs nontransfusion: outcome: cancer recurrence rates. Answer: Figure 2 legends were shown in 2–3 paragraph of page 7.

Five studies reported the cancer recurrence rate, 4478 participants for evaluation, which was higher in ABT group (32.9%, 416 of 1265 participants) than in nontransfused patients (12.3%, 395 of 3213 patients) (RR = 1.82, 95% CI 1.32–1.51, *P* = 0.0003) (Fig. 2).

The meta results after adjustment of covariates were shown in Table 4. The effect of age, sex, stage, tumor size, lymph node status, and histology were adjusted when pooled the data. Our results showed that ABT was an independent risk factor for cancer recurrence (RR = 0.672, 95% CI 0.567–0.796, *P* < 0.001).

### Postoperative Complication Rate

**FIGURE 3:**
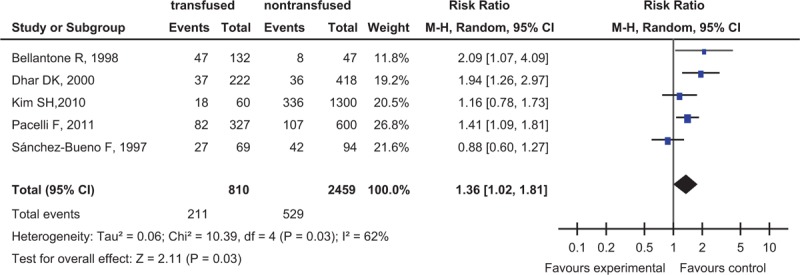
Forest plot of comparison: transfusion vs nontransfusion: outcome: postoperative complication rates. Answer: Figure 3 legends were shown in 4 paragraph of page 7.

Five studies reported the relationship between ABT and the postoperative complication rate, 3269 participants for evaluation, which was higher in ABT group (26%, 211 of 810 participants) than in nontransfused patients (21.5%, 529 of 2459 patients) (RR = 1.36, 95% CI 1.02–1.81, *P* = 0.03) (Fig. 3). The *P* value was greater than the threshold only when the data of complications were adjusted by meta-regression (RR = 0.713, 95% CI 0.508–1.001, *P* = 0.051) (Table 4), which would be discussed in the “Discussion” section.

### Subgroup Analysis

In the subgroup analysis, we found that 5-year survival rates were not associated with the transfusion rates (χ^2^ = 0.37, *P* = 0.54), even low transfusion rate increased the risk of death in transfused group, and the 5-year survival rate was lower in transfused group than in nontransfused group (RR = 0.73, 95% CI = 0.70–0.77, *P* < 0.00001). We also found that transfusion rates were higher in female than male patients and in stage of TNM III–IV group than Tis–II group as well (Table 2 and Table 3).

## DISCUSSION

In this meta-analysis, we found that ABT was associated with worse clinical outcome for patients undergoing GC surgery via the meta-analysis of the 16 cohort studies from 7 countries (3 continents, Asia, Europe, America), including 9189 participants, and the 5-year survival rate was lower for transfused patients than nontransfused patients; in subgroup analyses, the transfusion rate was higher in female than in male patients, which was a significant difference (χ^2^ = 24.41, *P* < 0.00001), transfusion rate increased significantly in patients of tumor stage TNM III–IV compared with Tis–II (χ^2^ = 90.02, *P* < 0.00001), and the 5-year survival rate had nothing to do with the transfusion rate, which indicated that even low transfusion rate could elevate the risk of death of patients with GC resection. In Table 4, the *P* value was greater than the threshold after adjusting the data of complications by meta-regression (*P* = 0.051) (Table 4), maybe some reasons the following, on the one hand, only 5 studies with patients’ complications were searched online, which enable the heterogeneity of complications to become some greater, on the other hand, the result was statistical differences in Figure 3, although not so significant, RR = 1.36, 95% CI 1.02–1.81, *P* = 0.03, so we believed it was necessary to give an advanced research next time, for example, to increase the number of searched studies, but we did not think that it would affect the total result of this meta-analysis.

It was thought that the adverse outcome of transfusion was related to the immune system because transfusion could downregulate hematopoiesis and subsequently downregulate the immune response.^18^ It was reported that perioperative surgical trauma and stress had an immunosuppressive impact on GC patients,^19^ and ABT exacerbated the impaired immune response,^20^ and remaining leukocytes in transfused blood played an important role in immunosuppression and transfusion-related immunomodulation,^21,22^ which could be some factors to result in tumor recurrence. Taylor et al^23^ reported that 1717 trauma patients in the intensive care unit, it was shown that nosocomial infections were 6 times higher and mortality was 2 times higher in the transfusion group than in the nontransfusion group. So, the patients who received transfusion developed a serious destroying in resistance to infections and cancer metastasis. We thought that the impaired immune system and the suppressing of hematopoiesis were contributed to the adverse clinical outcomes for patients with GC undergoing surgery.

In our meta-analysis, the survival rate was significantly lower; the incidence of cancer recurrence and postoperative complication was higher in transfusion group than in nontransfusion group. But the transfusion rate did not correlate with the survival rate; therefore, it was believed that transfusion itself mediated its effects on the survival rate rather than the transfusion rate, which was consistent with Kim et al.^1^ In this study, we also found that the transfusion rate was higher in female patients and patients of worsening stage (Table 3), which was consistent with Acheson et al,^24^ who reported that a meta-analysis of transfusion in colorectal cancer surgery patients, it was shown that the transfusion rate was higher in women than in men (OR = 1.15, 95% CI 1.06–1.24, *P* < 0.001).

The multivariate regression was used to analyze whether confounding factor besides transfusion had any effect on the 5-year survival rates, from the results of the multivariate regression survival analyses (Table 4), the transfusion was an independent risk factor on the survival rate of GC patients during preoperative period.

In our study, the transfusion rate was highly variable across studies ranging from 4.4% to 70.3%. Such large variations were also reported in other fields, for example, colorectal cancer surgery,^24^ total hip replacement surgery, total knee replacement surgery, and coronary artery bypass graft in 16 Austrian centers ranging from 16% to 87%, 12% to 87%, and 37% to 63%, respectively.^25^ There was not a consistent transfusion criterion in GC surgery patients; the overall evaluation should be done in patients before transfusion. We believe that it is important to restrict transfusion if possible; Furthermore, the establishment of a unanimous standard of the appropriate time for preoperative transfusion is urgently required.

## LIMITATIONS OF THIS STUDY

There were some limitations in this meta-analysis: first, there were some kinds of different products of transfusion, for example, some patients received erythrocytes, some received plasma, some received both of them, which we had not divided into relevant group; and second, some patients also suffered from other disease, but multiple reviewers had investigated the problem to minimize the extent of such bias, such as using multivariate regression to exclude the confounding factor, subgroup analysis to clarify the heterogeneity. We believed that it was unlikely to impact the clinical outcome by these biases.

## CONCLUSIONS

In conclusion, we found that it had a negative relation between ABT and prognosis in patients undergoing GC surgery, including the survival rates reduced, the risk of tumor recurrence and complications increased, the patients’ 5-year survival rate does not correlate with the transfusion rate, even low transfusion rate could elevate the mortality of patients with GC surgery.
